# Bioinformatics and Immune Infiltration Analyses Reveal the Key Pathway and Immune Cells in the Pathogenesis of Hypertrophic Cardiomyopathy

**DOI:** 10.3389/fcvm.2021.696321

**Published:** 2021-08-23

**Authors:** Xu-Zhe Zhang, Si Zhang, Ting-Ting Tang, Xiang Cheng

**Affiliations:** ^1^Department of Cardiology, Union Hospital, Tongji Medical College, Huazhong University of Science and Technology, Wuhan, China; ^2^Key Laboratory of Biological Targeted Therapy of the Ministry of Education, Wuhan, China

**Keywords:** hypertrophic cardiomyopathy, immune infiltration, biomarker, resident tissue macrophages, bioinformatics analyses

## Abstract

**Objective:** This study was designed to identify the key pathway and immune cells for hypertrophic cardiomyopathy (HCM) via bioinformatics analyses of public datasets and evaluate the significance of immune infiltration in the pathogenesis of HCM.

**Methods:** Expressional profiling from two public datasets (GSE36961 and GSE141910) of human HCM and healthy control cardiac tissues was obtained from the GEO database. After data preprocessing, differentially expressed genes (DEGs) were then screened between HCM and healthy control cardiac tissues in parallel. Gene Ontology, pathway functional enrichment, and gene set enrichment analysis were performed using DAVID and GSEA application. The compositional patterns of immune and stromal cells in HCM and control cardiac tissues were estimated based on the merged data using xCell. Protein–protein interaction (PPI) network and module analyses were constructed by STRING and Cytoscape applications. Gender-based expressional differences analyses were also conducted to explore gender differences in HCM. GSE130036 and clinical samples were used for verification analyses.

**Results:** A total of 310 DEGs were identified. Upregulated DEGs were mainly enriched in “adhesion” and “apoptotic process” in the biological process. As for the downregulated DEGs, “inflammatory response,” “innate immune response,” “phagosome,” and “JAK-STAT signaling pathway” were highly enriched. Immune infiltration analyses suggested that the scores of macrophages, monocytes, DC, Th1, Treg, and plasma cells in the HCM group were significantly decreased, while CD8^+^ T cells, basophils, fibroblasts, and platelets were significantly enriched. Module analyses revealed that STAT3, as the hub genes in HCM together with LYVE1^+^CD163^+^ macrophages, may play a key role in the pathogenesis of HCM while there were no obvious gender differences in the HCM samples from selected datasets. Verification analyses performed on GSE130036 and clinical samples showed a strong positive correlation (Spearman correlation = 0.7646) and a good co-localization relationship between LYVE1 and CD163, suggesting the potential function of LYVE1^+^CD163^+^ macrophages in maintaining the homeostasis of cardiac tissue.

**Conclusion:** STAT3-related pathway and CD163^+^LYVE1^+^ macrophages were identified as the potential key pathway and immune cells in HCM and may serve as interesting targets for further in-depth research.

## Introduction

Hypertrophic cardiomyopathy (HCM), as a complex genetic heterogeneous disease, is one of the main causes of sudden cardiac death in young adults (1). Epidemiological studies have shown that the prevalence rate of HCM in the general population is estimated to be 0.2% (2). The clinical characteristic of HCM is asymmetric cardiac hypertrophy. As for the cellular level, cardiac myocytes in HCM usually appear to be hypertrophied, disorganized, and separated by areas of fibrosis (3). Although MYH7 and MYBPC3, which encode beta-myosin heavy chain and myosin-binding protein C, respectively, are the two most common HCM-related mutant genes, a large number of underlying mutations and mechanisms are still undetermined (4).

Although regarded as a genetic disease, there is still a gap in how to fully understand the contribution of sarcomere gene mutation in the overall pathophysiological mechanism and clinical process of HCM. A wide range of opinions have suggested that the focus of HCM research should be shifted from single-gene hypotheses to alternative and complementary mechanisms, and novel determinants of HCM should be reviewed and identified in a comprehensive manner from the perspective of network medicine (5). The immune system can not only play the role of immune surveillance, maintaining normal physiological functions in the steady-state heart, but also mediate adverse inflammatory reactions and myocardial remodeling after injury (6–8). Studies have shown that the myocardium of HCM patients shows inflammatory cell infiltration and fibrosis (9). From the mechanism point of view, cardiac hypertrophy, as a pathological stimulus, will, in turn, cause inflammatory signal transduction and immune cell activation, thereby affecting heart function (7). It is still unclear whether this process will be independent or coordinated with the main pathogenic sarcomere mutation genes and thus interfere with the clinical course and prognosis of HCM patients. Above all, more extensive identification of key pathways and non-sarcomeric mutation-related pathophysiological mechanisms in HCM is very necessary and urgent.

Over the past decade, microarray, high-throughput sequencing techniques together with integrated bioinformatics analyses have provided tremendous assistance in identifying novel key genes and pathways in the pathological process of diseases. Here, we conducted full-sided bioinformatics analyses of public datasets to identify differentially expressed genes (DEGs) between HCM and healthy cardiac tissues, and explore the potential pathological mechanism of HCM via functional enrichment analysis including Gene Ontology (GO) and Kyoto Encyclopedia Genes and Genomes (KEGG) for evaluation, immune infiltration analysis, and protein–protein interaction (PPI) network construction. In addition, we explored the role of gender differences in HCM. Finally, we verified our conclusions through online dataset and clinical samples. We hope that our analyses can initially explore the potential role of signaling pathways and immune cells in HCM and provide useful targets for future in-depth research.

## Materials and Methods

### GEO Datasets

Gene expression profile data were collected from the Gene Expression Omnibus (https://www.ncbi.nlm.nih.gov/geo), a public database of NCBI that contains a mass of datasets for gene expressional profiling by arrays and high-throughput sequencing. We screened potential workable datasets based on the following searching criteria: “hypertrophic cardiomyopathy” MeSH Terms AND “Homo sapiens” Organism. Qualified datasets had to meet the following inclusion criteria: (i) Microarray or high-throughput sequencing profiling studies on human patients with HCM together with non-HCM control/healthy samples for comparison; (ii) reports of adequate sample sizes for analyses; (iii) group label for each sample size; and (iv) availability of the raw data. Finally, GSE36961 and GSE141910 were selected for analyses.

**Figure 1:**
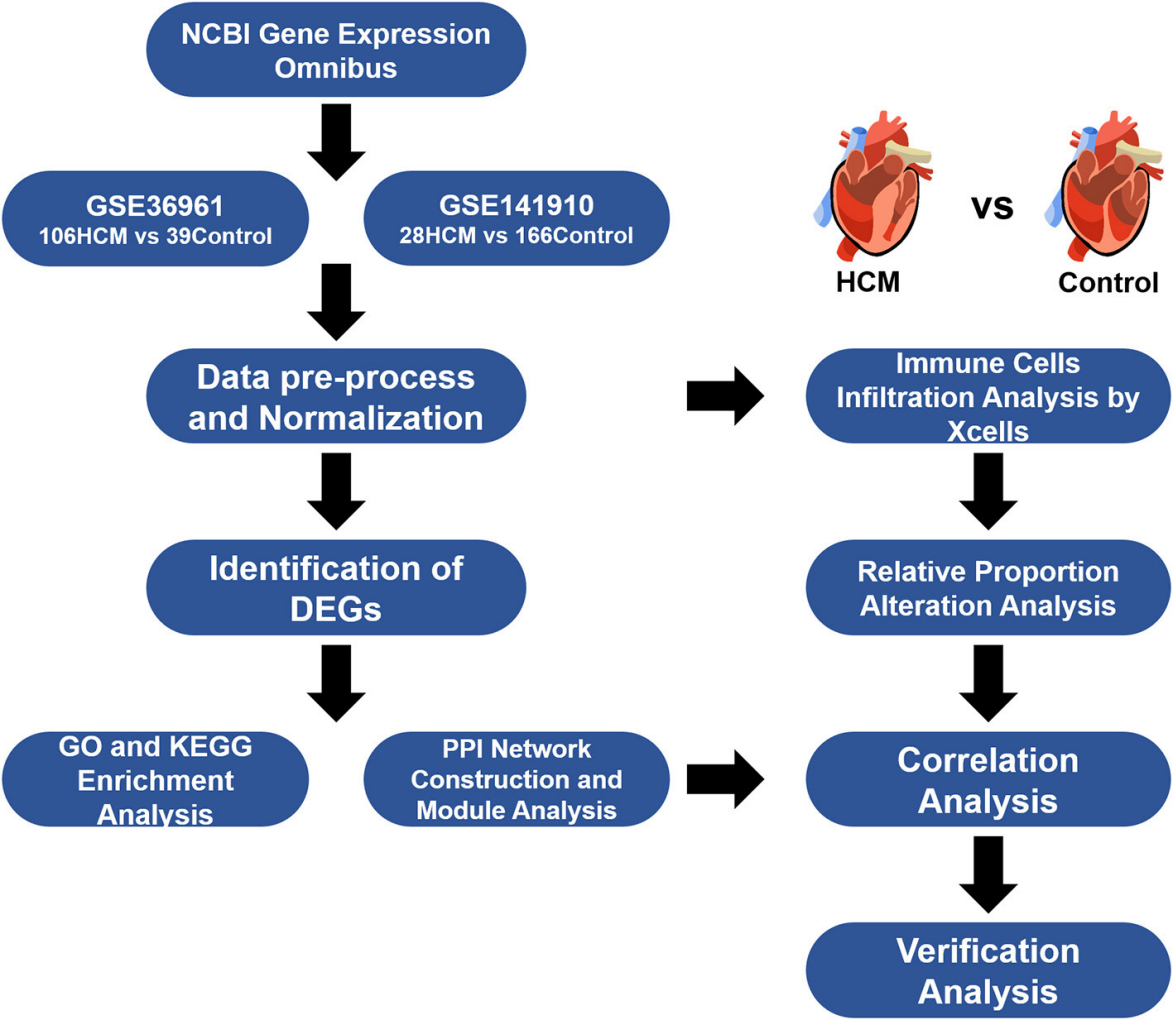
Workflow of the study.

HCM-affected myocardium (*n* = 106) from GSE36961 were collected from HCM patients undergoing therapeutic surgical septal myectomy for relief of obstructive symptom while control tissue (*n* = 39) consist of donor hearts without suitable transplant recipient. Clinical background for analyzed populations and detailed operation process in GSE36961 can refer to the original citation of this dataset (10) while a brief summary was listed in Supplementary Table 1. Data of GSE141910 came from the Myocardial Applied Genomics Network (www.med.upenn.edu/magnet). Left ventricular tissues in this series were harvested at the time of cardiac surgery from subjects with heart failure undergoing transplantation and control samples from unused donor hearts. Since the publication of GSE141910 has not yet been released, we can only use the information provided with the dataset and summarized the clinical background information of samples included as shown in Supplementary Table 2. Figure 1 provides an overview of our analyses workflow.

### Data Preprocessing and DEGs Identifying

The raw datasets were obtained from GEO and preprocessed by log2 transformation. Sample data were normalized by the limma package (11) in R software version 3.6.1 (https://www.r-project.org/) by plotting and adjusting the boxplot of each sample so as to maintain the data from a parallel experiment at the same level. The R package limma was also used for the identification of DEGs between different groups. *p*-values were adjusted by the Benjamini–Hochberg method. An adjusted *p* < 0.05 and a |log2FC| value >0.5 were applied in screening significantly different expression levels of molecules. Volcano plots and heat maps for DEGs were plotted using R packages “ggplot” and “ComplexHeatmap” under the platform of R software. The gene symbols of DEGs were recognized and mapped by FunRich software version 3.1.3 and Venn diagrams were drawn by the online plot tool Sangerbox (http://www.sangerbox.com/tool).

### GO, Pathway Functional Enrichment, and Gene Set Enrichment Analysis

GO and KEGG pathway enrichment analysis were performed using the DAVID (12) web service (https://david.ncifcrf.gov/) by inputting the DEGs. DAVID is an analytical platform that integrates multiple annotation datasets and could be used for the assignment of biological functions and potential pathways to the DEGs found to be up- and downregulated in HCM. The cutoff values for GO were set as *p* < 0.05 and KEGG with *p* < 0.1 analyses were set as *p* < 0.05. Dot plots for functional enrichment analyses were drawn by “enrichplot” packages under R software. Gene set enrichment analysis (GSEA) was conducted using the GSEA desktop application (13) in order to investigate enriched immune process-related signaling pathways. Visualization of GSEA results was performed using the Sangerbox tools.

### Immune Cell Infiltration Analysis

xCell (14) is a novel gene signature-based strategy used to infer variety immune and stromal cell types and is validated using extensive *in silico* simulations and also cytometry immune-phenotyping. Applying xCell to the normalized data, portrayals of cellular heterogeneity landscape for cardiac tissue expression profiles can be acquired. Several cell types were picked out and categorized based on their properties into four categories, namely, “lymphoid cells,” “myeloid cells,” “stromal cells,” and “stem cells and others.” Group violin graphs were plotted and evaluated by *t*-test to compare the cell types from the HCM group to healthy control. Cutoff values for significance were *p* < 0.05. Correlation matrix of immune cell subtypes and hub genes were constructed by “corrplot” and “psych” package in R. Pearson correlation coefficients were calculated and used for evaluating the strength of correlation.

### PPI Network Construction and Module Analysis

The identified DEGs were inputted and analyzed by the STRING (version 11.0) website service (15) with a minimum interaction score of 0.4. The networks were then exported and constructed by Cytoscape software (16), a platform for visualizing complex PPI networks. By applying the available Apps including cytoHubba (17) and MCODE (18) in Cytoscape, we identified hub gene sets by degree and recognized highly interconnected clusters as potential functional molecular complexes of HCM. Plots were directly output from Cytoscape. Pearson correlation analyses were performed for correlation between immune cells and hub genes. Identified clusters were then input into the TRRUST (19) database (https://www.grnpedia.org/trrust/) for transcription factor–target interaction information, and *p* < 0.05 was considered of significance.

### Verification of Crucial Genes and Cells in HCM

To verify the crucial genes in HCM, we downloaded another open strand-specific RNA-seq dataset GSE130036 from GEO containing expression profiles of myocardial tissues in 28 HCM patients and 9 healthy donors. HCM samples were harvested from septal myectomy while control hearts were collected from the left ventricle of unused healthy donated hearts. Clinical characteristics of patients could refer to the original publication (20) or Supplementary Table 3. Data were preprocessed based on the above criteria. Results were plotted by GraphPad Prism with *p*-value calculated through Welch's *t*-test, and the correlation plots were drawn using “ggplot2” package in R. Also, to examine the co-localization relationship between key gene and target immune cells in cardiac tissue, we conducted immunofluorescence staining on cardiac tissue harvested from multi-organ donors whose hearts were refused because of incipient coronary plaque formation in the left descending coronary artery. Heart tissues were then fixed in 4% paraformaldehyde at 25°C for 24 h, embedded in paraffin, and cut into 5-μm sections. A section from each heart was blocked with 1% BSA PBS buffer and stained with target fluorescent antibody. Finally, these sections were stained with DAPI to visualize the nuclei and viewed with confocal fluorescence microscopy. The protocol of the study was in accordance with the Declaration of Helsinki and was approved by the medical ethics committee of the Tongji Medical College of Huazhong University of Science and Technology (METC number: 2016S124).

### Statistical Analysis

Public data preprocessing and analyses including sample data merging, ID conversion, and duplication removal were performed using SPSS 22.0 and R software. *p* < 0.05 was considered statistically significant in all sections. Graphs were constructed with R software, GraphPad Prism, and online plot tools as described in the above sections in detail as well as the statistical methods used in this study.

## Results

### Identification of DEGs Between HCM and Healthy Cardiac Tissue

GSE36961 and GSE141910 were included in our bioinformatics analyses. GSE36961 had 145 samples, namely, 106 HCM and 39 healthy control cardiac tissues. GSE141910 had 28 HCM and 166 healthy control cardiac tissues. The HCM samples of the above two datasets came from the ventricular septum of patients with obstructive symptoms that require surgical intervention and the left ventricle of patients with heart failure, both of which represent the period when pharmacological treatment cannot control the symptoms of HCM, while the control samples were derived from unused healthy donated hearts; therefore, we considered these two datasets to be comparable. Considering that the two datasets included are of different sequencing types, the identification of DEGs was performed separately.

**Figure 2:**
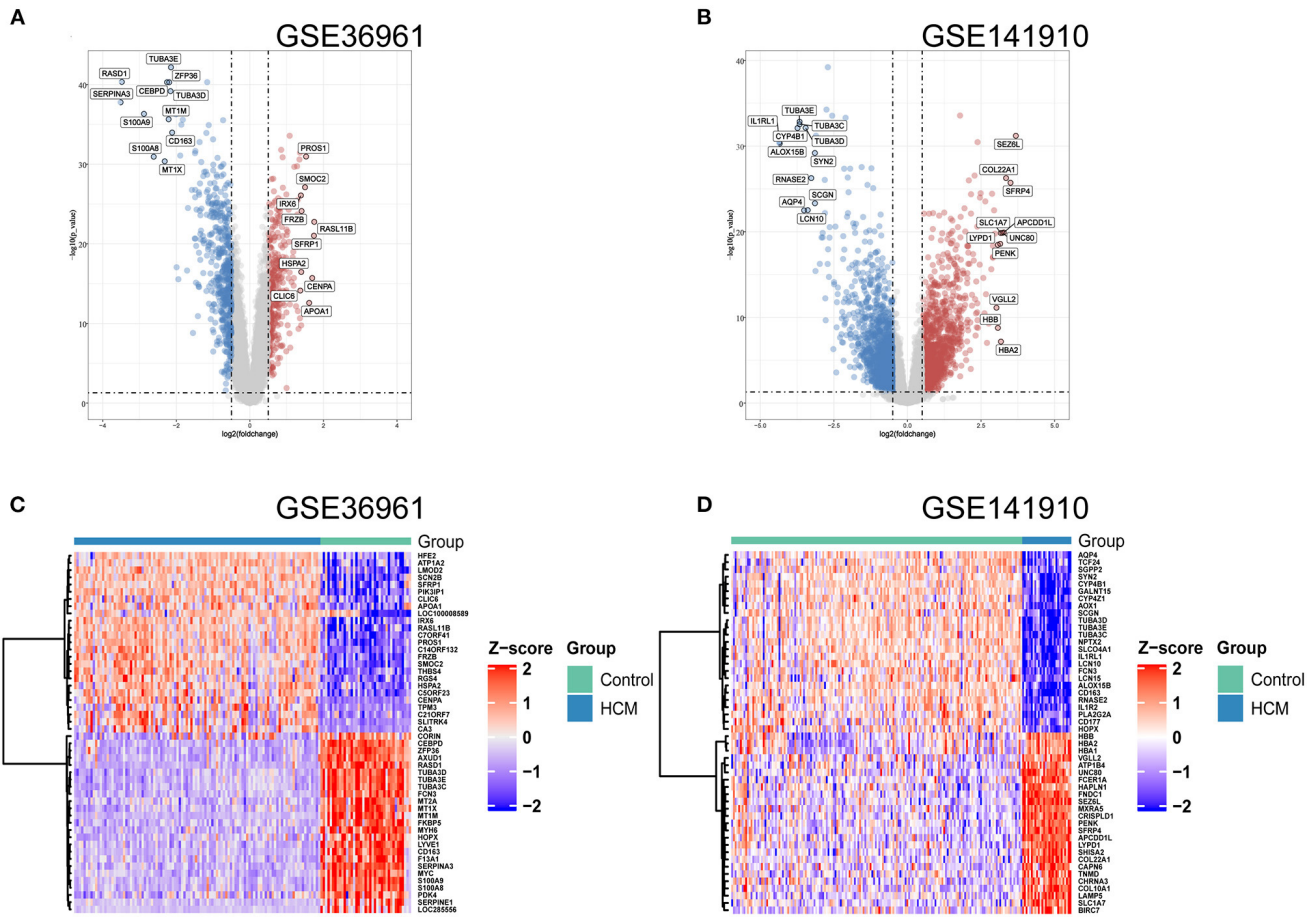
Differentially expressed genes (DEGs) of used datasets. **(A,B)** Volcano plot of DEGs between HCM and healthy control cardiac tissues in GSE36961 and GSE141910. **(C,D)** Heat maps of potential DEGs between HCM and healthy control cardiac tissues in GSE36961 and GSE141910.

**Figure 3:**
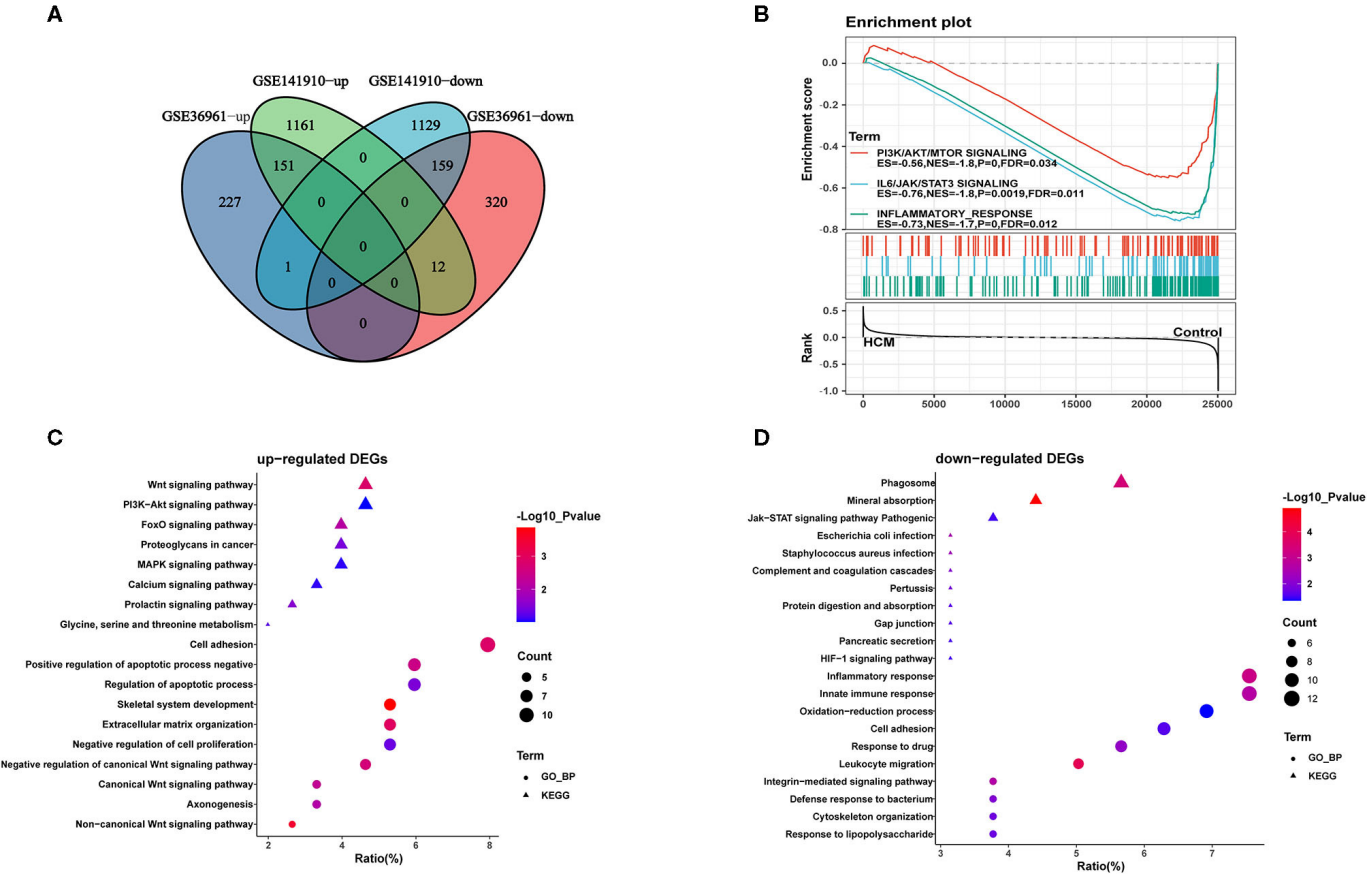
Identification and functional annotations of DEGs. **(A)** Venn diagram of up- and downregulated DEGs in GSE36961 and GSE141910 datasets. **(B)** GSEA for significantly enriched pathways in GSE36961. **(C,D)** GO and KEGG functional enrichment of up- and downregulated DEGs in HCM and healthy control cardiac tissues.

As shown in the volcano plots and heat maps in Figures 2A,C, 870 DEGs (379 upregulated and 491 downregulated) were recognized in the GSE36961. In addition, a total of 2613 DEGs (1324 upregulated and 1289 downregulated) were obtained from the GSE141910 dataset (Figures 2B,D). By taking the intersections of the Venn diagram in Figure 3A, we obtained 159 DEGs that were upregulated in HCM together with 159 downregulated DEGs from both datasets.

### Function and Pathway Annotations of DEGs

GO analyses were conducted to assess the consistency for functional annotations of the DEGs in the two datasets. Inputting all DEGs of the two datasets into DAVID, we obtained the functional annotation descriptions for DEGs of the two datasets. As shown in Supplementary Figure 1, two datasets shared a large degree of overlap in GO analysis including “calcium ion binding” and “heparin binding” in molecule function, “extracellular region” and “cell surface” in cellular component, and “inflammatory response” and “apoptotic process” in biological process, indicating good homogeneity and reliability between the two datasets.

Furthermore, we conducted GO-biological process and KEGG pathway analyses by inputting the up- and downregulated DEGs in DAVID separately. As shown in Figure 3C, upregulated DEGs were mainly enriched in “adhesion” and “apoptotic process” in the biological process together with Wnt and PI3K-Akt signaling pathway, which were significantly enriched. As for the downregulated DEGs in Figure 3D, “inflammatory response” and “innate immune response” were highly enriched; in addition, “phagosome” and “JAK-STAT signaling pathway” were also ranked high in KEGG. We then took data in GSE36961 and conducted GSEA analysis, and the results shown in Figure 3B suggested significant enrichment of inflammatory response and JAK-STAT signaling pathway in the control group.

### Immune and Stromal Cell Infiltration Analysis

**Figure 4:**
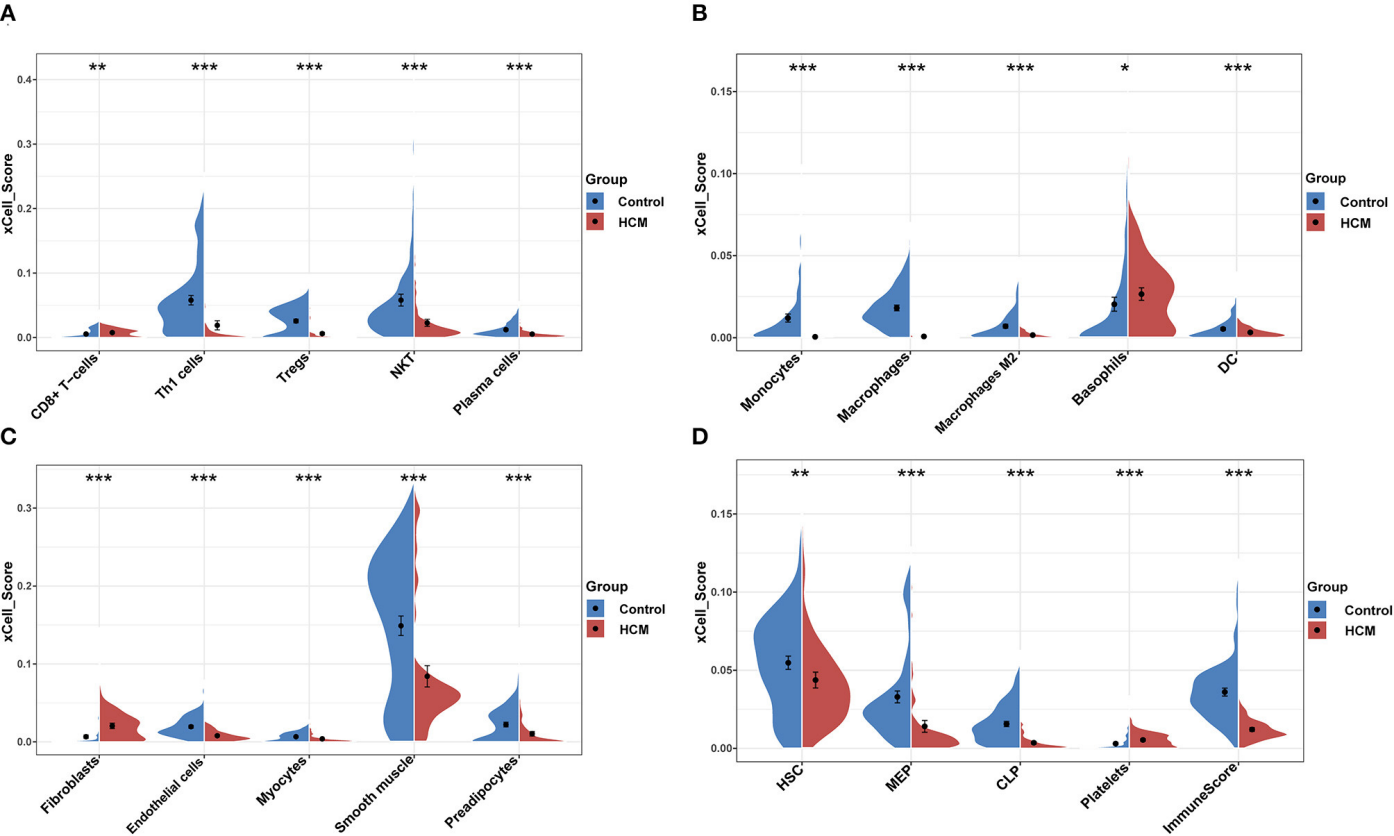
xCell scores of immune or stromal cells between HCM and healthy control cardiac tissues in GSE36961 and GSE141910. **(A–D)** Violin charts of “lymphoid cells,” “myeloid cells,” “stromal cells,” and “stem cells and others,” respectively. *p <0.05, **p <0.01, ***p <0.001.

xCell was used to identify immune or stromal cell types that may be involved in HCM. It generated enrichment scores of different cell types by using a large number of gene expression data. Because the xCell scores were not affected by different sequencing types, we combined the scores of GSE36961 and GSE141910 for further analysis. We divided 64 kinds of cells into four subsets, namely, “lymphoid,” “myeloid,” “stromal,” and “stem cells and others.” There were 26 cell types with significant differences in HCM cardiac tissue vs. control group in the merged dataset as partly shown in Figures 4A–D, among which the scores of macrophages, monocytes, DC, Th1, Treg, and plasma cells in HCM group were significantly decreased, while CD8^+^ T cells, basophils, fibroblasts, and platelets were significantly enriched.

**Figure 5:**
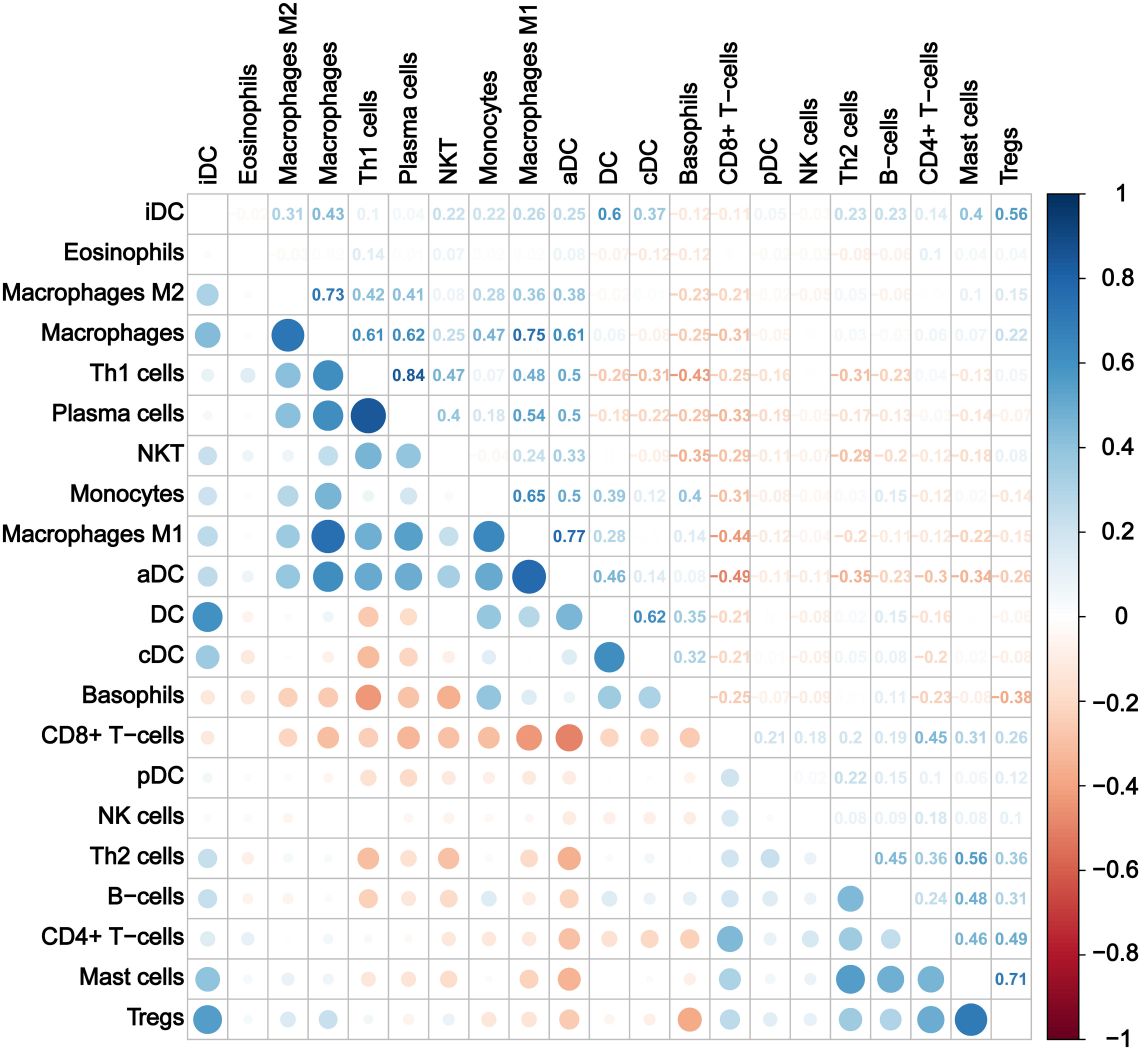
Correlation matrix of immune cell subtypes.

Furthermore, we calculated the correlation efficient between each immune cell to explore their relationship and possible interaction. As shown in Figure 5, among all immune cells, Th1 had the highest correlation with plasma cells (Pearson's correlation = 0.84), and the second strongest positive correlation was the correlation between macrophage M1 and aDC (Pearson's correlation = 0.77). In addition, macrophages as a whole showed strong positive correlation with both macrophages M1 and M2. On the other hand, CD8^+^ T cells and aDC showed the strongest negative correlation (Pearson's correlation = −0.49).

### PPI Network Construction and Module Analysis

**Figure 6:**
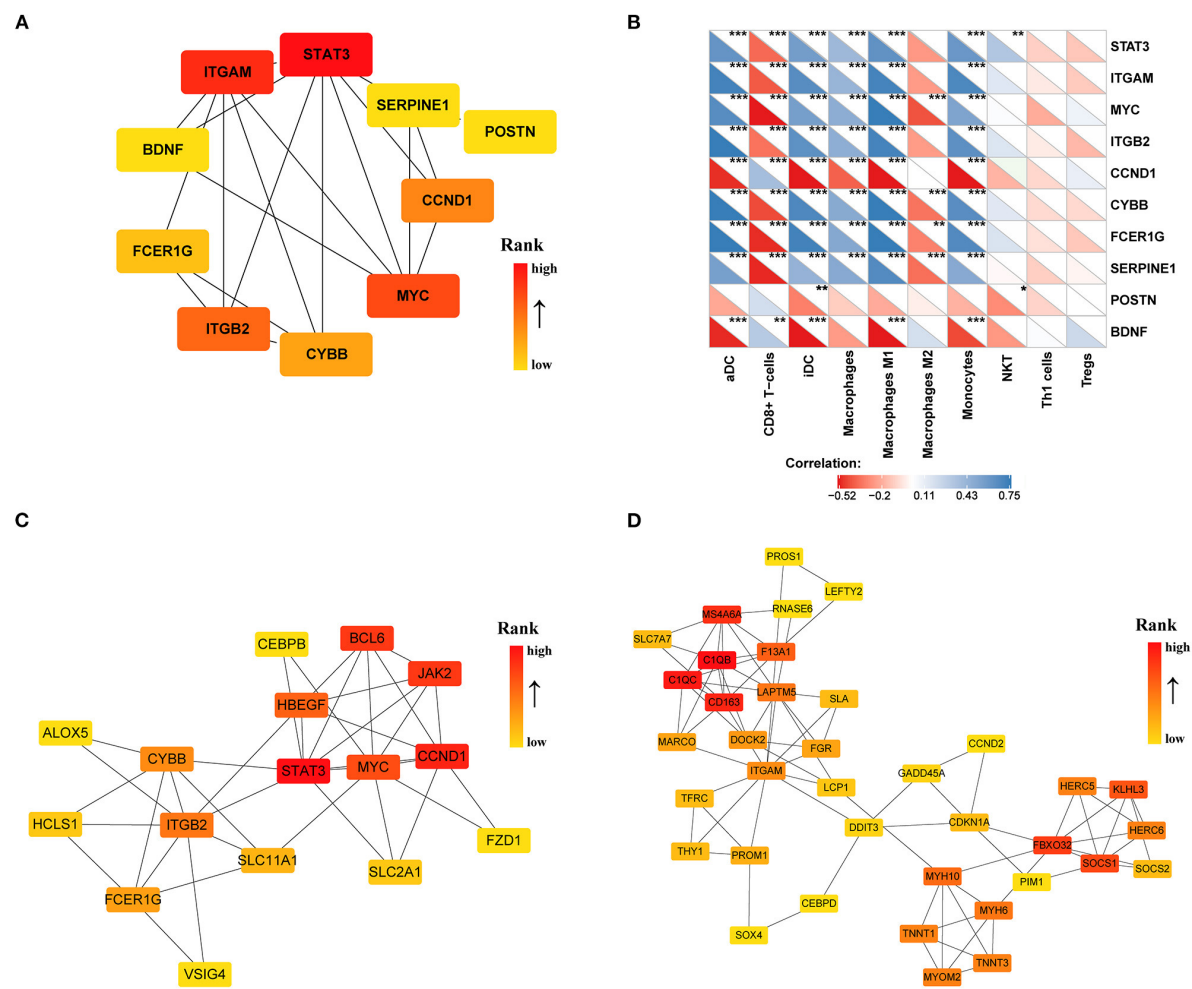
PPI network of DEGs (|log2FC| >0.5) in the GSE36961 and GSE141910 dataset. **(A)** Top 10 hub genes in DEGs. **(B)** Correlation matrix of xCell scores of immune cell subtype with top 10 hub genes. **(C,D)** Two highly interconnected clusters recognized by MCODE in DEGs. *p <0.05, **p <0.01, ***p <0.001.

To identify the significant pathways of HCM, we uploaded the filtered 308 DEGs into the STRING and obtained the exported TSV file for the interactions of multiple DEGs to Cytoscape for further network construction. CytoHubba identified 10 hub genes (Figure 6A), among which only BDNF, CCND1, and POSTN were upregulated in HCM; all the rest were downregulated. We then calculated the correlation efficient between 10 hub genes and interested immune cell types. As shown in Figure 6B, macrophages M1, iDC, aDC, monocytes, and CD8^+^ T cells presented a strong and reliable correlation to most of the hub genes, suggesting that the key genes in the DEGs had a great effect on the positive regulation of immune system. MCODE then recognized two highly interconnected clusters in DEGs as shown in Figures 6C,D. We used TRRUST to predict key candidate regulators for cluster 1 and cluster 2, and results are listed in Supplementary Tables 4, 5. For both clusters, STAT3 (Signal transducer and activator of transcription 3) was predicted to be the key regulator. In addition, for cluster1, HIF1A, and TP53 seemed to play a crucial role while ABL1 and NFYA ranked high in the prediction of cluster2.

**Figure 7:**
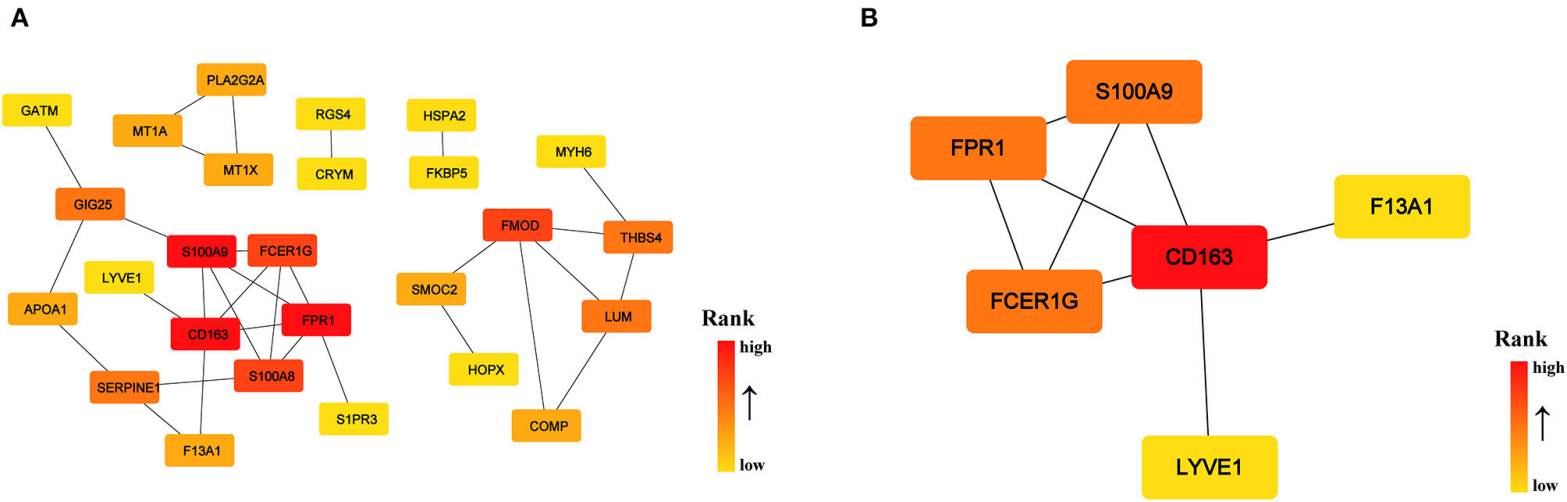
PPI network of significant DEGs (|log2FC| >1.0) in the GSE36961 and GSE141910 dataset. **(A)** PPI network of significant DEGs. **(B)** Cluster recognized by MCODE in significant DEGs.

To identify the key genes of HCM that were highly related to the function of immune cells, we filtered the 308 DEGs with |log2FC| value >1.0 and obtained 51 DEGs of significant difference. Further PPI network construction, hub genes, and clusters identification shown in Figures 7A,B suggested that CD163, regarded as a highly specific marker of macrophages M2, played the most important role in the downregulated DEGs while FMOD, the key gene in upregulated DEGs, was mainly related to the process of fibrosis. The CD163-related genes including FPR1, S100A9, FCER1G, LYVE1, and F13A1 were identified to be the candidate key genes highly related to the function of macrophages in HCM. Referring to the information on GeneCards, we summarized the relationships between candidate key gene with immune cells. As shown in Table 1, S100A9, FCER1G, and F13A1 were reported to be widely expressed in different kinds of immune cells while FPR1 was mainly expressed in neutrophils. We then focused on LYVE1 and its function in HCM together with its relationship with macrophages M2.

**Table 1:** Relationships between candidate key genes with immune cells ( Log_2_FC from GSE36961/GSE141910).

**Gene symbol**	**Log_**2**_FC^†^**	**Adj. *p*-value**	**Related immune cells**
CD163	−2.113/−2.704	<0.001	Monocytes
FPR1	−1.367/−1.313	<0.001	Neutrophil/Monocytes
S100A9	−2.879/−1.534	<0.001	Neutrophil/Monocytes/Lymphocytes
FCER1G	−1.482/−1.020	<0.001	Monocytes/Lymphocytes
LYVE1	−1.863/−2.002	<0.001	Monocytes
F13A1	−1.577/−1.179	<0.001	Neutrophil/Monocytes/Lymphocytes

† *represents the annotation in the title box*.

### Gender Differences in HCM

To explore the potential role of gender differences in HCM, we selected samples of HCM patients from two datasets and performed differential expression analysis according to gender. Obtained DEGs between male and female HCM patients are shown in Supplementary Table 6. Most DEGs are related or linked to sex chromosomes. In addition, MYL4 (myosin light chain 4) was found to be upregulated in male HCM patients from GSE36961, NPPA (Natriuretic Peptide A) was upregulated in female patients, and VIT was upregulated in male patients from GSE141910.

Furthermore, we examined whether gender differences would affect the expression of CD163 and LYVE1 in cardiac tissues of HCM patients. Still, we used the expressional matrix of GSE36961 and GSE141910 to calculate the relative expression differences of CD163 and LYVE1 between male and female. As shown in Supplementary Figure 2, there seems to be no gender difference in the expression of CD163 and LYVE1 in HCM patients.

### Verification of Crucial Genes and Immune Cells in HCM

**Figure 8:**
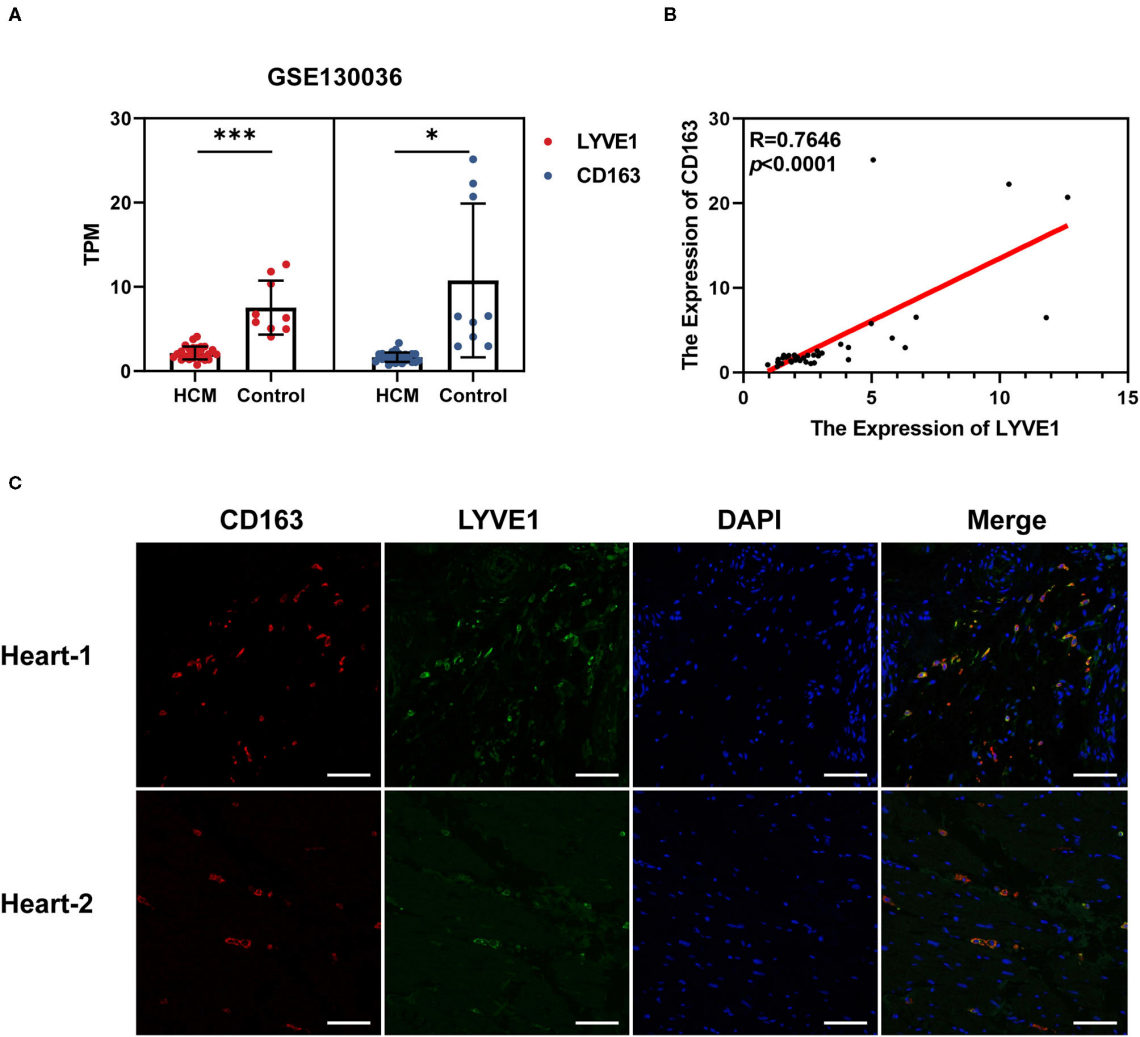
Verification of crucial genes and immune cells in HCM. **(A)** Expression profiles of CD163 and LYVE1 in the verification dataset GSE130036. **(B)** Correlation plot of CD163 and LYVE1 in the verification dataset GSE130036. **(C)** Representative fluorescent staining images of heart-infiltrating CD163^+^ and LYVE1^+^ macrophages from cardiac tissues of two healthy donors (scale: 100 μm). *p <0.05, **p <0.01, ***p <0.001.

We chose GSE130036 as the validation dataset to verify the expression profiles of CD163 and LYVE1 between HCM and healthy control cardiac tissue. As shown in Figures 8A,B, CD163 and LYVE1 were significantly low expressed in HCM while the expression profiles of these two genes show a strong positive correlation (Spearman correlation = 0.7646). The immunofluorescence staining results in Figure 8C presented a good co-localization relationship between LYVE1 and macrophage M2. Above all, these results verified our previous analysis and suggested that the dysfunction of LYVE1^+^ CD163^+^ macrophages plays a vital role in the progression of HCM.

## Discussion

The specific underlying pathogenesis of HCM, one of the main causes of sudden death in young adult, is still unclear. More and more studies have shown that cardiovascular diseases, such as myocardial infarction (MI) and heart failure, are often accompanied with abnormal pro-inflammatory activation of the immune system and disorders of the cardiac-resident immune cells (21). Exploring the mechanism of the key immune cells and pathways in the pathogenesis of cardiomyopathy can clarify the specific role of the immune system in the maintenance and imbalance of cardiac function to a certain extent, so as to provide potential therapeutic targets on future cardiovascular immunotherapy.

It is well-known that macrophages have strong plasticity and will be polarized in response to different environmental stimuli during development (22). A widely used paradigm is defined as “classical” activated macrophages M1 and “alternative” activated macrophages M2 based on their function (23). Macrophages M1 are usually pro-inflammatory cells involved in the initial inflammatory reaction, while macrophages M2 have anti-inflammatory effects and are closer to the characteristics of steady-state resident tissue macrophages (RTM) (24, 25). Macrophage is the most important member of cardiac immune cells. In the healthy heart of adult mice, macrophage accounts for about 5–10% of the total number of non-cardiomyocytes, while in the resident CD45^+^ immune cells, it accounts for up to 80% (26). With the deepening of research, the biological functions of cardiac RTM continue to expand. In a healthy heart, cardiac RTM is involved in promoting the development and maturation of coronary arteries in the embryonic stage (27), promoting atrioventricular node electrical conduction (28), and maintaining myocardial mitochondrial homeostasis (29). After the heart of newborn mice is injured, the RTM in it inhibits bad ventricular remodeling by promoting the proliferation of cardiomyocytes and the formation of blood vessels (30). In adult MI model mice, removal of cardiac RTM resulted in worsening of heart function and bad ventricular remodeling (31). Therefore, cardiac RTM not only participates in the maintenance of healthy heart tissue homeostasis but also participates in the repair of the heart after injury. Considering the significant number of macrophages in cardiac tissue together with its important organ-specific functions, it is necessary to further explore their phenotypes, functions, and dynamic changes between steady-state cardiac with disease-state.

LYVE1 (lymphatic vessel endothelial receptor-1) is one of the main receptors of hyaluronic acid in the body. Previous studies have found that LYVE1 is highly expressed on the basal surface and inner surface of lymphatic endothelial cells, and it participates in regulating the entry of immune cells from tissues into lymphatic vessels by combining with the hyaluronic acid coating on the surface of inflammatory cells (32). Deletion of Lyve1 in mice will prevent entering and traffic of leukocytes through the lymphatic endothelium, causing worsening of chronic inflammation and long-term deterioration of cardiac function (32). Recently, based on single-cell transcriptome analysis, Chakarov et al. found two cardiac RTM subgroups in mice identified by LYVE1^low^ and LYVE1^high^. LYVE1^low^ cardiac RTM is mainly distributed around nerve fibers with the strong ability of antigen presentation, while LYVE1^high^ cardiac RTM is mainly distributed around capillaries with possible anti-fibrosis function (33). Together, the existing research results have already hinted to us the possibility that LYVE1^+^ macrophages may serve as homeostatic markers in cardiac tissue. Our results further suggest that the reduction of CD163^+^LYVE1^+^ macrophages may be an important process for the pathogenesis of HCM; it may act as a bridge between the heart's immune system and the lymphatic system to maintain the steady-state function of the heart. The process of pathological myocardial hypertrophy could mediate cardiac dysfunction and long-term damage by interfering with this connection, thereby affecting the clinical symptoms and prognosis of HCM patients.

STAT3 is one of the members of the STAT family and can be expressed in various tissues and organs, including myocardium (34). In recent years, a number of studies have found that STAT3 and its related signaling pathways play a key regulatory role in the pathophysiological processes of cardiovascular system, such as myocardial hypertrophy (35), ischemia–reperfusion injury (36), and myocardial fibrosis (37). Several lines of evidence have indicated that members of the IL-6 family are able to induce cardiomyocyte hypertrophy via gp130-mediated STAT3 activation while deletion of IL-6 weakened transverse aortic constriction-induced ventricle hypertrophy together with marked attenuation of STAT3 activation (35). Our analysis results suggest that more in-depth research is needed to explore the relationship between STAT3-related pathways and the pathogenesis of HCM, so as to provide ideas to further search for related treatment strategies.

Gender difference in HCM has always been an attractive perspective. Studies have shown that female HCM patients are generally older, have more severe obstructive symptoms than male, and are at higher risk of progression to heart failure or death (38). However, the specific mechanism of this phenomenon is still unclear. We grouped HCM samples by gender and performed differential expression analysis and found that DEGs were mainly related or linked to sex chromosomes, while MYL4 and VIT were found to be slightly upregulated in the cardiac tissue of male patients, and NPPA was upregulated in female. It is reported that the percentage of MYL4-positive cells in male ventricular myocardium is higher, and it is also moderately related to HCM (39). However, there is still no evidence to prove the specific mechanism of gender regulating MYL4 expression in ventricular tissues. NPPA is often considered to be a marker of cardiac hypertrophy, and its elevated levels may be related to worsening cardiac function (40). The level of NPPA in the ventricular tissue of female HCM patients was significantly higher than that of males, suggesting that the female patients analyzed had a higher degree of deterioration of cardiac function, which was similar to the aforementioned clinical statistical results. VIT (vitrin) encodes an extracellular matrix (ECM) protein and its connection with HCM and gender has not yet been reported, and further exploration is needed. We also explored whether there is a difference in the expression of CD163 and LYVE1 in HCM patients of different genders, and the results showed that there is no significant difference in the expression of the two genes between male and female. Based on the above analysis, we temporarily believe that the differences in the clinical manifestations and prognosis of HCM between male and female may be more related to differences in diagnosis and clinical pathways caused by social, genetic, and endocrine factors, but still, more in-depth research on gender differences in HCM patients is needed.

In this study, we analyzed the GSE36961 and GSE141910 datasets related to HCM. A total of 339 samples are included to comprehensively analyze the state of immune cell infiltration in HCM and the correlation between immune cells. In addition, we built a network to screen for genes that play a key role in HCM and validated the results by verifying the dataset GSE130036 and the collected clinical samples for immunofluorescence analysis, which made our research results more reliable. In addition, the impact of gender differences in HCM was also explored, but we have not obtained any novel findings. In all, our results indicate the potential important roles of STAT3-related signaling pathway and LYVE1^+^CD163^+^ macrophages in the pathogenesis of HCM. It is worth noting that CD163, as a typical marker of macrophages M2, exhibits a good co-localization and a strong positive correlation with LYVE1. We speculate that the decrease in the proliferation of CD163^+^LYVE1^+^ macrophages or the decrease in the number caused by phenotypic conversion is a key part of the pathogenesis of HCM. It is necessary to study the specific mechanism of the role of this macrophage subset in cardiac hypertrophy, and the development of immunotherapy targeting RTM may help to alleviate heart injury and promote the recovery of heart function.

However, our study has certain limitations. The most important thing is that our study was based on bioinformatics immune infiltration analysis from the transcriptomic profiles of public datasets, which may be in discordance with actual scenarios. Secondly, whether there is a causal relationship between gene expression differences and the pathogenesis of HCM or whether it was just modified by compensatory mechanisms cannot be clearly determined. Finally, due to the difficulty of obtaining cardiac samples from HCM patients, we only verified the co-localization relationship between LYVE1 and CD163 in normal donated hearts, instead of comparing and quantifying the difference in the number of CD163^+^LYVE1^+^ macrophages in the cardiac tissue of HCM patients and healthy donors.

## Conclusion

In summary, through bioinformatics analyses of public transcriptome data, STAT3-related pathway and CD163^+^LYVE1^+^ macrophages were identified as the potential key pathway and immune cells in HCM and may serve as interesting targets for further in-depth research.

## Data Availability Statement

The datasets presented in this study can be found in online repositories. The names of the repository/repositories and accession number(s) can be found in the article/Supplementary Material.

## Ethics Statement

The studies involving human participants were reviewed and approved by medical ethics committee of the Tongji Medical College of Huazhong University of Science and Technology. The patients/participants provided their written informed consent to participate in this study.

## Author Contributions

X-ZZ and SZ conducted statistical analysis, carried out the experiments, and drafted the article. T-TT contributed to reviewing the article. XC edited and revised the article. All authors contributed to manuscript revision, read, and approved the submitted version.

## Conflict of Interest

The authors declare that the research was conducted in the absence of any commercial or financial relationships that could be construed as a potential conflict of interest.

## Publisher's Note

All claims expressed in this article are solely those of the authors and do not necessarily represent those of their affiliated organizations, or those of the publisher, the editors and the reviewers. Any product that may be evaluated in this article, or claim that may be made by its manufacturer, is not guaranteed or endorsed by the publisher.
