# Supplementary material for: Bioinformatics and Immune Infiltration Analyses Reveal the Key Pathway and Immune Cells in the Pathogenesis of Hypertrophic Cardiomyopathy
Source: Front Cardiovasc Med. 2021 Aug 23;8:696321. doi: 10.3389/fcvm.2021.696321 (PMC8419431; doi:10.3389/fcvm.2021.696321)
Supplement: Supplementary file 1 [file Data_Sheet_1.pdf]

Bioinformatics and Immune Infiltration Analyses Reveal Key Pathway and Immune Cells in The Pathogenesis of Hypertrophic Cardiomyopathy

Supplementary Material

Xu-Zhe Zhang[1,2†], Si Zhang[1,2†], Ting-Ting Tang[1,2] and Xiang Cheng[1,2*]

[1]Department of Cardiology, Union Hospital, Tongji Medical College, Huazhong University of Science and Technology, Wuhan 430022, China

[2]Key Laboratory of Biological Targeted Therapy of the Ministry of Education, Wuhan 430022, China

1 Supplementary Figures

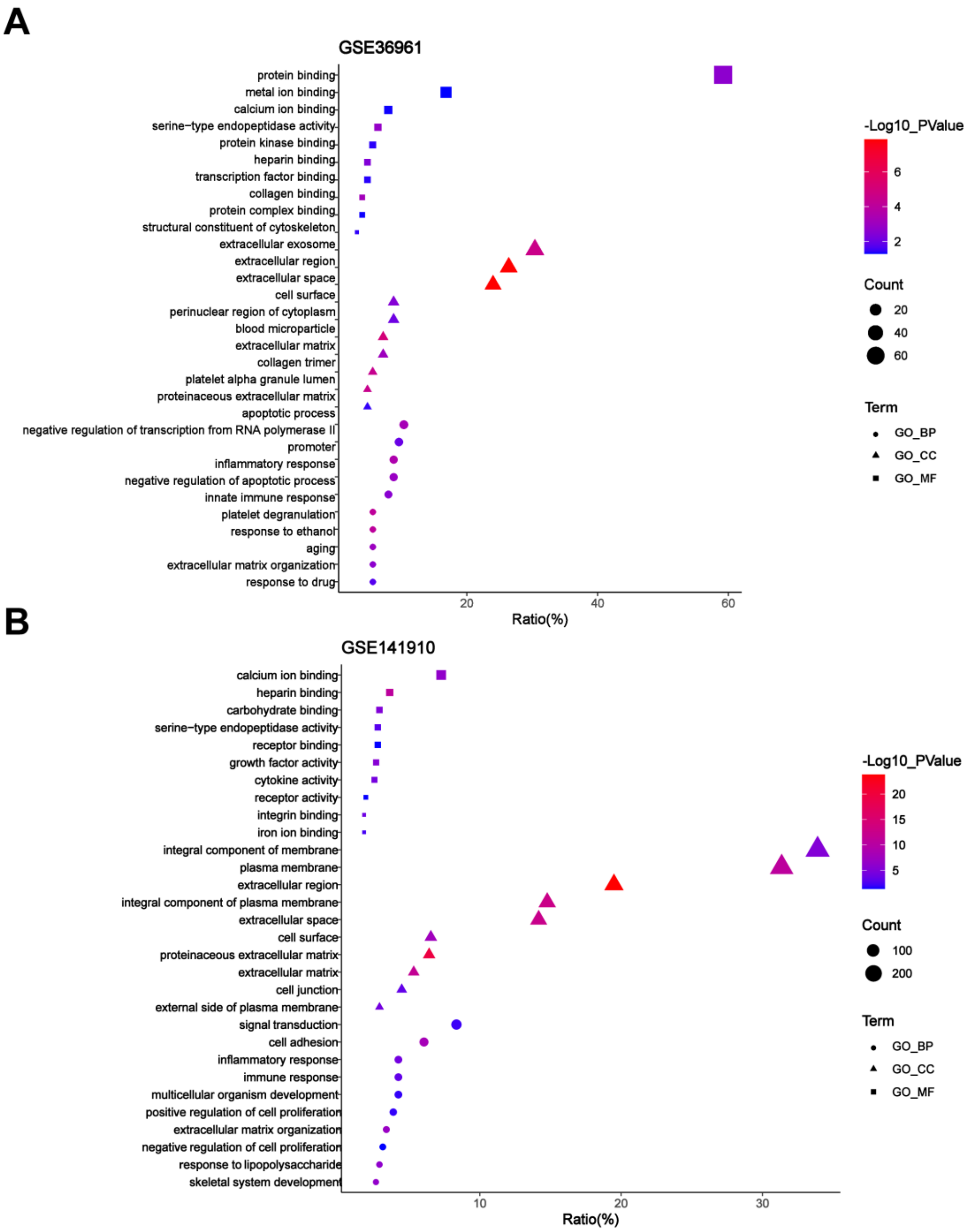

Supplementary Figure 1. Functional annotations of DEGs in GSE36961 and GSE141910 respectively. (A) GO and KEGG functional enrichment of DEGs in HCM and healthy control cardiac

tissues in GSE36961. (B) GO functional enrichment of DEGs in HCM and healthy control cardiac tissues in GSE141910.

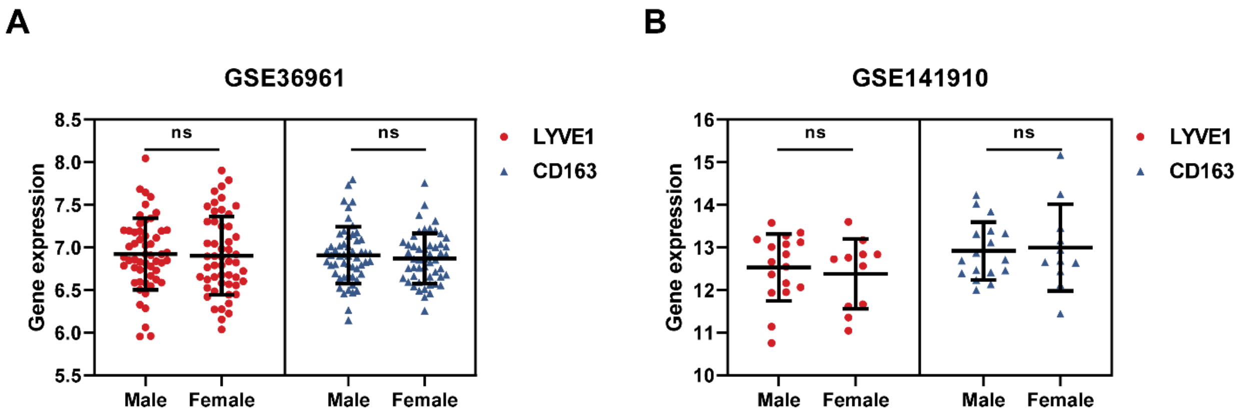

Supplementary Figure 2 gender difference in the expression of CD163 and LYVE1 in HCM patients. (A) GSE36961. (B) GSE141910.

2 Supplementary Tables

Table S1 Clinical characteristics of involved samples in GSE36961.

	Cases	Controls
No.	106	39
Sex (male/female), no.	54/52	19/20
Age, surgery (y), median (IQR)	51(32-60)	NA
NYHA class III-IV, no. (%)	82 (77)	NA
Ejection fraction (%), median (IQR)	74 (68-77)	NA
Sample source	Left ventricular septum	Left ventricular septum

Table S2 Clinical characteristics of involved samples in GSE141910.

	Cases	Controls
No.	28	166
Sex (male/female), no.	17/11	77/89
Age, surgery (y), median (IQR)	49(41-57)	56(51-65)
Sample source	Left ventricle	Left ventricle

Table S3 Clinical characteristics of involved samples in GSE130036.

	Cases	Controls
No.	28	9
Sex (male/female), no.	19/9	8/1
Age, surgery (y), median (IQR)	33(28-40)	NA
LVEF (%)	71(67-75)	NA
Sample source	Left ventricular septum	Left ventricular septum

Table S4 Candidate key regulators of cluster1 predicted by TRRUST.

Key TF	# of overlapped genes	P.Value	FDR
HIF1A	5	6.12e-09	2.57e-07
BRCA1	4	1.32e-07	1.98e-06
SPI1	4	1.87E-07	1.98E-06
TP53	5	1.88E-07	1.98E-06

BCL6	3	3.38E-07	2.84E-06
ESR1	4	4.26E-07	2.98E-06
CTCF	3	2.00E-06	1.20E-05
RELA	5	3.77E-06	1.98E-05
STAT3	4	5.21E-06	2.43E-05
EP300	3	1.35E-05	5.39E-05

Table S5 Candidate key regulators of cluster2 predicted by TRRUST.

Key TF	# of overlapped genes	P.Value	FDR
STAT3	6	3.13E-07	9.69E-06
ABL1	3	8.25E-07	1.28E-05
NFYA	3	7.73E-06	7.99E-05
POU5F1	3	1.37E-05	0.000106
JUND	3	3.98E-05	0.000221
MYC	4	4.28E-05	0.000221
IRF9	2	0.000104	0.00046
IRF1	3	0.000135	0.000525
BRCA1	3	0.000135	0.000525
BCL6	3	0.000189	0.00065

Table S6 DEGs in HCM patients grouped by gender.

Gene Symbol	$Log_2FC^{\dagger}$	adj.P.Value	Gene name
EIF1AY*	5.194/10.206	<0.001	Eukaryotic Translation Initiation Factor 1A Y-Linked
RPS4Y1*	4.599/9.512	<0.001	Ribosomal Protein S4 Y-Linked 1
JARID1D/ KDM5D*	2.062/9.617	<0.001	Lysine Demethylase 5D (Y-Encoding)
RPS4Y2*	1.470/NA	<0.001	Ribosomal Protein S4 Y-Linked 2
TTTY14*	1.226/NA	<0.001	Testis-Specific Transcript, Y-Linked 14
TXLNGY*	1.086/NA	<0.001	Taxilin Gamma Pseudogene, Y-Linked
DDX3Y*	NA/9.842	<0.001	DEAD-Box Helicase 3 Y-Linked
VIT	NA/1.113	<0.05	Vitrin
MYL4	0.978/NA	<0.01	Myosin Light Chain 4
USP9Y*	0.842/9.378	<0.001	Ubiquitin Specific Peptidase 9 Y-Linked
PRKY*	0.699/NA	<0.001	Protein Kinase Y-Linked
NPPA	-1.380/NA	<0.05	Natriuretic Peptide A
XIST*	-2.245/NA	<0.001	X Inactive Specific Transcript

† Log_2FC from GSE36961/GSE141910, “+” for up-regulated in male, “-” for up-regulated in female

* for gender-related or linked gene
